# Genogroup-Specific Multiplex Reverse Transcriptase Loop-Mediated Isothermal Amplification Assay for Point-of-Care Detection of Norovirus

**DOI:** 10.3390/diagnostics15151868

**Published:** 2025-07-25

**Authors:** Wahedul Karim Ansari, Mi-Ran Seo, Yeun-Jun Chung

**Affiliations:** 1 Department of Medical Sciences, The Catholic University of Korea, Seoul 06591, Republic of Korea; ansari23@catholic.ac.kr; 2 Department of Microbiology, The Catholic University of Korea, Seoul 06591, Republic of Korea; 3 Precision Medicine Research Center, The Catholic University of Korea, Seoul 06591, Republic of Korea; 4 Department of Microbiology and Public Health, Patuakhali Science and Technology University (Barishal Campus), Barishal 8210, Bangladesh; 5 Research and Development Department, Connectagen Inc., Hanam 12918, Republic of Korea

**Keywords:** norovirus, genogroups, RT-LAMP, gastroenteritis

## Abstract

**Background/Objectives:** Norovirus is a major cause of acute gastroenteritis worldwide. Considering its highly infectious and transmissible nature, rapid and accurate diagnostic tools are of utmost importance for the effective control of outbreaks in the context of point-of-care testing (POCT). In this study, we developed a genogroup-specific multiplex reverse transcriptase loop-mediated isothermal amplification assay to detect the human norovirus genogroups I and II (GI and GII, respectively). **Methods:** For the comprehensive detection of clinically relevant genotypes, two sets of primers were incorporated into the assays targeting the RdRp-VP1 junction: one against GI.1 and GI.3, and the other for GII.2 and GII.4. Following optimization of the reaction variables, we standardized the reaction conditions at 65 °C with 6 mM MgSO_4_, 1.4 mM dNTPs, 7.5 U WarmStart RTx Reverse Transcriptase, and Bst DNA polymerase at 8 U and 10 U for GI and GII, respectively. Amplification was monitored in real-time using a thermocycler platform to ensure precise quantification and detection. Finally, the assay was evaluated through portable isothermal detection device to test its feasibility in on-site settings. **Results:** Both assays detected the template down to 10^2^–10^3^ copies per reaction and showed high target selectivity, yielding no non-specific amplification across 39 enteric pathogens. These assays enabled prompt detection of GI within 10–12 min and of GII within 12–17 min after the reaction was initiated. Onsite validation reveals all template detection below 15 min, demonstrating its potential feasibility in point-of-care applications. Including the sample preparation time, test results were obtained in less than 1 h. **Conclusions:** This method is a rapid, reliable, and scalable solution for detecting human norovirus in POCT settings for both clinical diagnosis and public health surveillance.

## 1. Introduction

Human Norovirus (HuNoV), a member of the Calciviridae family, is a single-stranded RNA virus that is the leading cause of acute gastroenteritis globally [1]. Approximately 685 million cases occur annually, with general symptoms of vomiting, abdominal cramps, and diarrhea. Although healthy individuals often experience self-limiting recovery, severe complications can arise, particularly in children, older adults, and immunocompromised hosts [2,3]. NoV is highly infectious and transmissible chiefly via the fecal–oral route, either directly through contact with an individual or indirectly through contaminated water, food, or surfaces [2,4]. Despite the World Health Organization (WHO) designating the development of NoV vaccines a high priority, there remains a lack of effective NoV vaccines owing to the inadequacy of robust cell culture tools and animal models [5]. Moreover, low infectious doses of NoV, environmental stability, and asymptomatic viral shedding make prevention difficult [6]. Therefore, there is a critical need for the accurate and rapid detection of NoV in the field to control outbreaks and mitigate their impact.

The NoV genome is extremely diverse, containing 10 genogroups and 48 genotypes. Genogroups I, II, and IV (GI, GII, and GIV, respectively) infect humans [7], and GI and GII are the predominant strains responsible for most clinical cases (approximately 90%) [3,8]. Differentiating NoV genogroups aids epidemiological surveillance, enabling swift interventions by public health authorities to control outbreaks. This further highlights the need to develop genogroup-specific assays as effective measures against NoV. Among conventional diagnostic techniques, such as electron microscopy, enzyme immunoassays, and reverse transcription PCR (RT-PCR), RT-PCR-based assays are the gold standard for NoV detection [9,10]. However, PCR-based methods require sophisticated machines and long running times, making them unsuitable for point-of-care testing (POCT) settings and limiting their effectiveness during epidemic outbreaks [11]. To address these challenges, loop-mediated isothermal amplification (LAMP) is a promising alternative solution for the prompt and highly specific detection of NoV. LAMP is a nucleic acid amplification technique that enables isothermal amplification of the target product [12]. The LAMP technique uses four to six primers that target eight precise sites on the template. These primers initiate the amplification process performed by DNA polymerase, which has a strand-displacement ability [12,13]. Therefore, large numbers of amplicons are produced within a short time, which can be detected using several methods, including colorimetric or fluorescent detection, turbidity measurement, gel electrophoresis, UV detection, and even real-time detection in a thermocycler [14,15]. These unique features are particularly beneficial for point-of-care applications, because they can be achieved using less equipment and basic infrastructure [12,16]. RNA can also be detected using LAMP, which incorporates a reverse transcriptase and DNA polymerase [12].

In conjunction with its numerous advantages and versatile detection methods, LAMP is an excellent option for the molecular diagnosis of different pathogens, particularly in resource-limited settings and under time constraints during a crisis response. Despite the benefits of LAMP technology, few studies have been conducted on its use for detecting NoV [17,18]; therefore, it is increasingly being applied to NoV for quick and precise detection. Moreover, as NoV has multiple genotypes and variants within each genogroup, detecting multiple genotypes and variants in a single reaction is of the utmost importance. Based on these factors, this study aimed to develop a genogroup-specific multiplex RT-LAMP technique for the enhanced detection of NoV.

## 2. Materials and Methods

### 2.1. Primer Design for NoV RT-LAMP Assay

**Figure 1:**
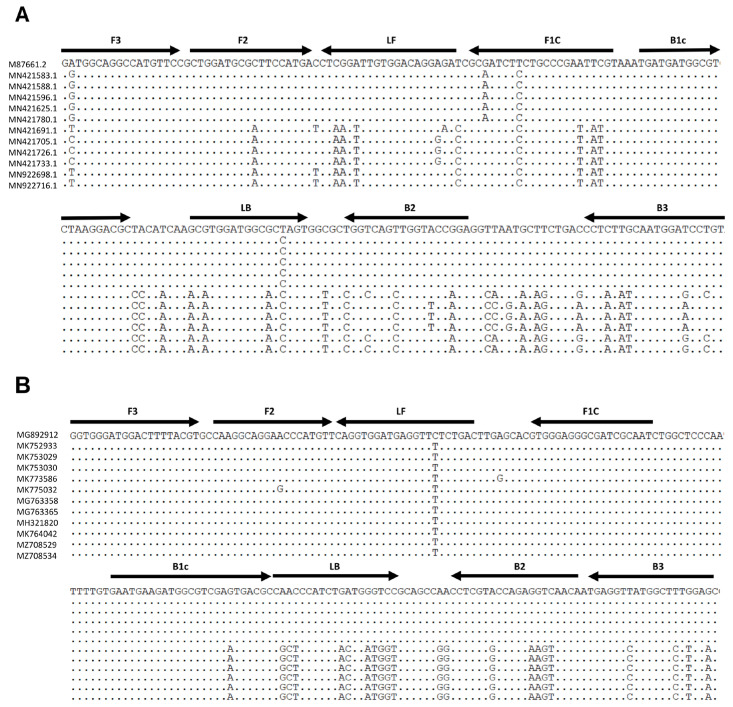
Multiple sequence alignment of the NoV RdRp-VP1 junction (BLAST retraction) and design of RT-LAMP primers. (**A**) Genogroup I (GI.1 and GI.3). (**B**) Genogroup II (GII.2 and GII.4). Shared regions across genotypes targeted by multiple primers (e.g., B3-1, B3-2) are labeled once (e.g., B3). Abbreviations: F3; forward outer primer, B3; backward outer primer, FIP [F2-F1C]; forward inner primer, BIP [B1C-B2]; backward inner primer, LF; loop forward primer, LB; loop backward primer, RdRp; RNA-dependent RNA polymerase, VP1; major capsid protein.

To design LAMP primers to detect NoV, the sequences of the most prevalent genotypes at the clinical stages (GII.4, GII.2, GI.3, and GI.1) were utilized [3]. Overall, 100 RNA-dependent RNA polymerase (RdRp) and capsid protein (VP1) gene sequences (50 for each genogroup) were retrieved from the GenBank database http://www.ncbi.nlm.nih.gov/genbank/ (accessed on 20 November 2024) and aligned using BioEdit version 7.2 software https://bioedit.software.informer.com/7.2/ (accessed on 21 November 2024). To design the LAMP primers, Primer Explorer version 5.0 https://primerexplorer.eiken.co.jp/lampv5e/index.html (accessed on 27 November 2024) was used [19], and the software initially produced the primers in the standard format, in which each primer set comprised two inner primers (forward inner primer [FIP] and reverse inner primer [BIP]), two outer primers (forward outer primer [F3] and reverse outer primer [B3]), and two loop primers (forward loop primer [LF] and reverse loop primer [LB]) to accelerate the reaction kinetics. However, as there were sequence variations among genotypes (GI.1 vs. GI.3) and (GII.2 vs. GII.4) additional primers were designed through manual curation and the incorporation of degenerate bases. These custom-designed primers bind to the same target regions and are sequence optimized to accommodate genotype-specific variations. The feasibility of all of the primer sets was assessed and confirmed by validation using BLAST version 2.16.0 (https://blast.ncbi.nlm.nih.gov/Blast.cgi) (accessed on 10 December 2024). The details of the primer sets used for NoV RT-LAMP are listed in Table 1. The primer binding sites were identified using BLAST-based multiple sequence alignment (Figure 1).

### 2.2. RNA and Plasmid Preparation

HuNoV standard RNAs for GI (ATCC 3234SD) and GII (ATCC 3235SD) were obtained from the American Type Culture Collection (ATCC; Manassas, VA, USA). These RNAs were used to validate the effectiveness of the designed primers, following the procedure outlined in the first RT-LAMP assay developed for NoV [17]. For plasmid construction, the most conserved region across the RdRp-VP1 junction was identified using multiple sequence alignment. Target sequences corresponding to GI.1, GI.3, GII.2, and GII.4 were synthesized and inserted into the pMG-Amp vector. The accuracy of all constructed plasmids was verified using sequencing (Macrogen, Seoul, Korea). The plasmid was reconstituted according to the manufacturer’s instructions and used as a template for the LAMP reaction.

### 2.3. RT-LAMP Assay

The RT-LAMP assay was performed as described previously [12,15,20]. Briefly, 25 uL of reaction mixture containing 2.5 uL of 10× isothermal amplification buffer (New England Biolabs, Ipswich, MA, USA), 6 mM MgSO_4_ (New England Biolabs), 1.4 mM of deoxynucleotide triphosphate (New England Biolabs), 8 U of Bst 2.0 DNA polymerase (New England Biolabs), 7.5 U of WarmStart RTx Reverse Transcriptase (New England Biolabs), 1.25 uM SYTO9 (Thermo Fisher Scientific, Waltham, MA, USA), and 2 uL of template plasmid DNA containing the RdRp-VP1 gene were mixed with the remaining volume of distilled water (DW). The RT-LAMP primer mix consisted of 0.2 uM of the outer primers (F3 and B3), 1.6 uM of the inner primers (FIP and BIP), and 0.4 uM of loop primers (LF and LB). The amplification reaction was initially carried out at 65 °C for 3 min, 1 cycle and followed by 90 subsequent cycles of 30 s each (total 48 min) at 65 °C using a CFX96 Touch Real-Time PCR detection system (Bio-Rad Laboratories, Hercules, CA, USA). The 90 cycles employed here are due to technical feasibility to enable real-time monitoring and data visualization on this platform, not an indication of thermal cycling.

### 2.4. Optimization of the NoV RT-LAMP Reaction

To optimize the conditions for efficient amplification, the RT-LAMP reaction was conducted under various conditions. We designed four primer sets for each genotype. For GI, primer set 1 included four inner primers (FIP-1, FIP-2, BIP-1, and BIP-2) and three outer primers (F3, B3-1, and B3-2). Set 2 incorporated set 1 with two additional forward loop primers (LF-1 and LF-2). Set 3 included two loop backward (LB-1 and LB-2) primers along with those in set 1. Finally, set 4 contained those in set 1 along with four loop primers (LF-1, LF-2, LB-1, and LB-2). For GII, primer set 1 included three inner primers (FIP, BIP-1, and BIP-2) and three outer primers (F3, B3-1, and B3-2). Set 2 included those in set 1 with an additional LF. Set 3 included two loop backward (LB-1 and LB-2) primers along with those in set 1. Finally, set 4 contained those in set 1 along with three loop primers (LF, LB-1, and LB-2). Different reaction temperatures (55 °C, 60 °C, and 65 °C), varying concentrations of Bst DNA polymerase (6 U, 8U, and 10 U), different dNTPs concentrations (1.2 mM, 1.4 mM, and 1.6 mM), and varying levels of MgSO_4_ (4 mM, 6 mM, and 8 mM) were applied in the reactions to determine the optimal conditions for the novel NoV RT-LAMP assays. To confirm the absence of unintended amplification, nuclease-free molecular-grade water was used as a negative control.

### 2.5. Sensitivity and Specificity of the NoV RT-LAMP Assays

The detection limit of the RT-LAMP assay was determined to assess its sensitivity. The template plasmid DNA was serially diluted ranging from 10^6^ to 10^2^ copies/reaction while RNA standards were diluted from 10^5^ to 10^2^ copies/reaction based on the initial concentration provided by the standard stock, and they were used in each real-time RT-LAMP assay. Experiments were conducted in triplicate for each template concentration. To determine the specificity of the RT-LAMP assay, the reactions were performed using nucleic acids from different enteric pathogens. Details of the pathogens are summarized in Supplementary Table S1.

### 2.6. Application to Point-of-Care Platform

To bring true point-of-care adaptability, we tested our assays using a portable isothermal amplification device called AnyDetect (Connectagen Inc., Korea). The device is lightweight (~900 g), compact (150 × 169 × 89 mm), battery-powered, and it detects nucleic acid amplification based on a turbidity measurement at 650 nm. Reaction mixture was the same, except no intercalating dye (SYTO9) was used. Additionally, 15 uL of mineral oil was added with reaction mixture to maintain reaction stability.

## 3. Results

### 3.1. Optimization of NoV RT-LAMP Conditions

**Figure 2:**
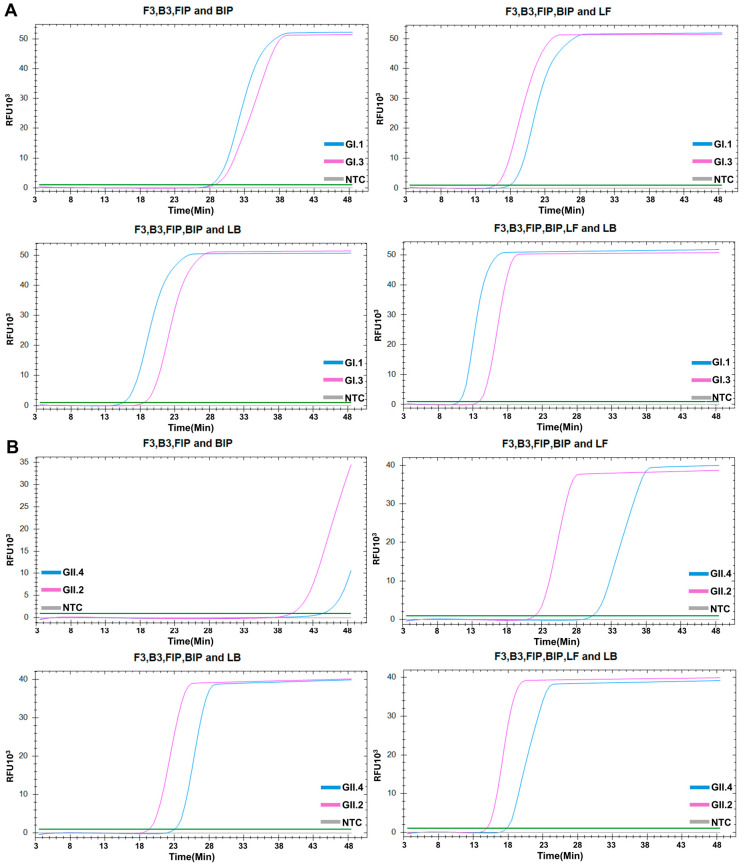
Optimization of the RT-LAMP primer sets for genogroup I (**A**) and genogroup II (**B**) amplification. Set 1: outer primers (F3 and B3) and inner primers (FIP and BIP); set 2: set 1 plus loop forward primer (LF); set 3: set 2 plus loop backward primer (LB); set 4: set 1 plus both loop primers (LF and LB).

We designed LAMP primers for the simultaneous detection of the major genotypes of NoV GI (GI.1, GI.3) and NoV GII (GII.2, GII.4). To achieve optimal assay performance, we designed four primer sets for each genogroup and compared their performance. In the context of different primer sets, set 4 showed the fastest amplification performance and identified targeted genotypes for both NoV GI (Figure 2A) and GII (Figure 2B); all negative controls remained negative throughout the assays. In addition, to validate the RT efficiency, we also performed RT-LAMP using RNA standards of Norovirus GI and GII obtained from ATCC and confirmed compatible amplification performance (Supplementary Figure S1).

**Figure 3:**
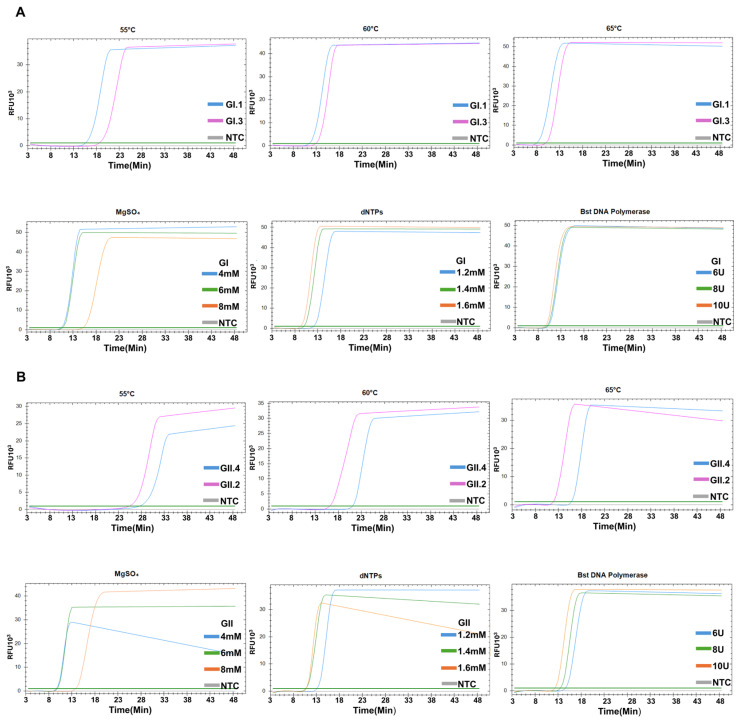
Optimization of RT-LAMP conditions for genogroup I (**A**) and genogroup II (**B**) amplification using different reaction temperatures and various concentrations of MgSO_4,_ dNTPs, and Bst DNA polymerase.

Therefore, the set 4 primers were used to develop RT-LAMP assays under different conditions. Next, we evaluated three reaction temperatures (55 °C, 60 °C, and 65 °C) to determine the optimal temperature for the assays, and 65 °C produced the most robust and consistent amplification in both genogroups (Figure 3A,B). Thus, 65 °C was selected as the standard temperature for subsequent reactions. Various concentrations of MgSO_4_ (4 mM, 6 mM, and 8 mM), dNTPs (1.2 mM, 1.4 mM, and 1.6 mM), and Bst DNA polymerase (6 U, 8 U, and 10 U) were also evaluated to assess the optimal RT-LAMP reaction conditions. Regarding MgSO_4_, 6 mM MgSO_4_ produced the highest amplification levels for both GI and GII. Regarding dNTP, 1.4 mM dNTPs showed the best results. Consequently, the final standard conditions were 65 °C, 6 mM MgSO_4_, 1.4 mM dNTPs, with 8 U of Bst DNA polymerase for GI (Figure 3A) and 10 U for GII (Figure 3B).

### 3.2. Sensitivity and Specificity of NoV RT-LAMP Assays

**Figure 4:**
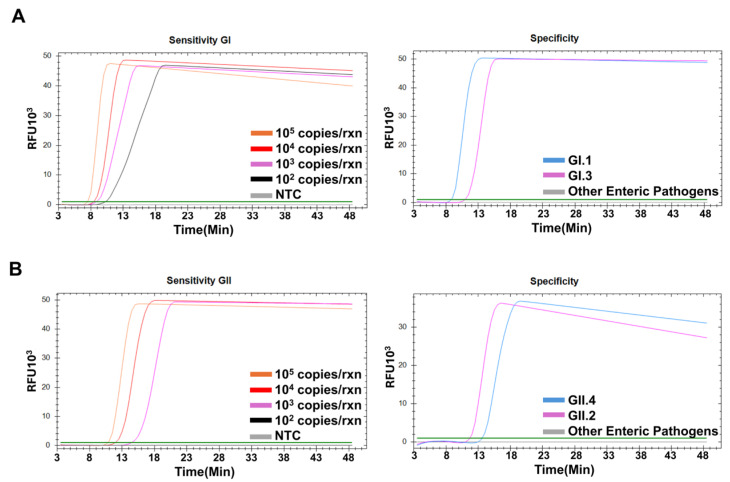
Sensitivity and specificity of the RT-LAMP assays. Serial dilutions of the standard RNA, ranging from 10^5^ to 10^2^ copies/reaction of genogroup I (**A**) and genogroup II (**B**) subjected to assays. For specificity, cross-reactivity was tested against 39 enteric pathogens.

The detection sensitivity of the NoV RT-LAMP assay was assessed using serial dilutions of the standard RNA and target plasmid DNA. The lowest concentration for GI standard RNA was 10^2^ copies per reaction, while GII was 10^3^ copies per reaction (Figure 4A,B). On the other hand, plasmid templates were detected as low as 10^2^ copies/reaction (Supplementary Figure S2). To evaluate specificity, a panel of 39 pathogens responsible for enteric infections was tested. Only the GI- and GII-specific positive controls showed positive amplification, whereas the other assays did not display detectable signals, confirming the specificity of this assay (Figure 4A,B).

### 3.3. Application of RT-LAMP Assay Under POCT Platform

To evaluate potential field applicability of our assays, we further tested using an isothermal amplification device as described in the methodology. All the target plasmids corresponding to GI.1, GI.3, GII.2 and GII.4 were successfully detected below 15 min of reaction initiation (Supplementary Figure S3A). To verify whether the RT step is also functioning in the portable device, we tested the standard RNAs (described in methodology section). Both standard RNAs were successfully amplified within 07 min, highlighting the assay’s potential for field deployment (Supplementary Figure S3B).

## 4. Discussion

Norovirus is a notable universal public health concern due to its widespread enteric outbreaks, heightened risk to vulnerable populations, and substantial economic impact [21]. Although PCR-based technologies are currently the gold standard for NoV detection, they remain unsuitable for POCT settings because of their complexity and substantial equipment requirements. RT-LAMP could be a promising alternative for rapid detection because of its speed, specificity, and minimal equipment requirements, which are beneficial during outbreaks [18]. In this study, we aimed to develop genogroup-specific multiplex RT-LAMP assays that cover the major genotypes of NoV (GI.1 and GI.3 in NoV GI and GII.2, and GII.4 in NoV GII), thereby making them highly suitable for POCT.

The RdRp-VP1 junction was selected to design LAMP primers. This junction is known to be highly conserved within each norovirus genogroup whilst exhibiting sufficient divergence between genogroups, making it ideal for developing genogroup-specific detection assays and enhancing the reliability of the assay [17,22]. To avoid nonspecific amplification during multiplex detection, in silico evaluation and experimental validation were performed, whilst maintaining a standard molar ratio of outer, inner, and loop primers (1:8:2) throughout the assay. Specificity was further confirmed by validating numerous pathogens. Similar approaches have been used to detect NoV and other enteric pathogens, such as *Vibrio parahaemolyticus*, where the inclusion of multiple primers increased detection speed and sensitivity [23,24].

We tested different reaction temperatures and concentrations of the pivotal elements. In our assays, optimal amplification was achieved at 65 °C. Although few studies have reported the use of RT-LAMP for NoV, two previous research groups detected GI and GII NoV at 62 °C [17,24]. In the experiments for optimizing MgSO_4_, both 4 mM and 6 mM yielded comparable amplification efficiency, but 6 mM was implemented as a standard because of its consistently higher RFU values and overall amplification robustness. Similarly, 1.4 mM and 1.6 mM dNTPs demonstrated similar results. However, 1.4 mM was preferred as the optimal concentration to limit reagent use and minimize the potential hazards associated with higher dNTP levels. Maximum reaction efficiency was achieved with different concentrations of Bst DNA polymerase. Therefore, 8 U was selected for GI and 10 U for GII. A comparison of optimal conditions for NoV RT-LAMP assays with those used for other pathogens revealed that the concentrations of MgSO_4_, dNTPs, and Bst DNA polymerase played comparable roles in ensuring maximal amplification [15,20,25]. Therefore, we established universal standard reaction conditions for both RT-LAMP assays of isothermal incubation at 65 °C with 6 mM MgSO_4_, 1.4 mM dNTPs, 7.5 U WarmStart RTx Reverse Transcriptase, and Bst DNA polymerase at 8 U for GI and 10 U for GII in a reaction volume of 25 uL. The LOD of the optimized RT-LAMP assay was 10^2^–10^3^ copies per reaction. Our sensitivity rate is comparable to that of previously published reports, which reported a similar detection limit [17,26]. Oysters and other seafood, which are major reservoirs of NoV, can be contaminated by as low as 100 genome copies [27], whereas NoV-infected individuals can shed viruses at approximately 10^9^–10^10^ copies/gram of stool [28,29]. Therefore, considering the detection limits, our assays are highly suitable for clinical and environmental samples. The analytical specificity of the RT-LAMP assay was assessed by examining a panel of 39 enteric pathogens, which revealed no cross-reactivity or false amplification. Previous studies have also shown good specificity, but they only covered about six enteric viruses, which does not represent the range of organisms that can be found in clinical and environmental settings [17,30]. In contrast, our broad coverage provides the most comprehensive specificity analysis using RT-LAMP for NoV detection to date, making it highly specific in terms of differential diagnosis in POCT settings. One of the key strengths of our assay is its ultrafast detection time, achieving results within 10–12 min for NoV GI and 12–17 min for NoV GII. This makes our assays superior to previous assays where 60 min or more was needed for detection without gel running [17,18,30].

Moreover, the integration of our RT-LAMP assays into a portable isothermal device significantly enhances their potential for field adaptability and POCT use. On the portable device, amplification detected within 7 min for standard RNA and 15 min for all the constructed plasmids, demonstrating fast and reliable performance under POCT conditions. It is worth mentioning that one research group, [30], achieved simultaneous detection of Norovirus genogroups GI and GII in a single reaction, but compared to their system our assay is more field adaptable due to it detecting within a very short time while their system needs 60–90 min. Unlike their gel-based detection, our method is fully compatible with portable devices, making it more suitable for POCT settings [30]. To date, different isothermal amplification systems have been applied to detect different pathogens. Recombinase polymerase amplification (RPA) assay is another isothermal nucleic acid amplification technology which works by mixing recombinase enzymes, single-stranded binding protein, and a strand-displacing DNA polymerase [31]. A team of researchers used RT-RPA to detect NoV [32]. According to the technical comparison of isothermal amplification technologies, both LAMP and RPA assays showed excellent sensitivity and specificity, however, the estimated cost for RPA was more than 10 times higher than LAMP mainly due to the enzyme cost [33]. Although we did not technically compare ours and Han et al.’s, they only targeted genogroup II whereas our system covers both GI and GII to achieve majority coverage of outbreak strains. Furthermore, we detected the target below 15 min in POCT settings, faster than RT-RPA technology. Taking these aspects together, our system would be suitable for NoV detection in the field.

Despite the significant features of our RT-LAMP assay, several limitations should be acknowledged. Firstly, the assay was validated using plasmid DNA and standard RNA, and so clinical or environmental sample testing in field conditions remains to be explored. Secondly, the assay was validated using important clinical genotypes. However, it has not been tested against several other genotypes which are less common and additional testing is needed to confirm full genogroup detection.

In conclusion, we developed a rapid, precise, and field-friendly RT-LAMP assay for norovirus GI and GII detection. With amplification detectable around 15 min and compatibility with portable devices, this assay offers a promising solution for real-time point-of-care diagnostics. Due to its strategic focus on the predominant circulating genotypes, the assay ensures practical applicability in the current medical field. Further validity with clinical samples will enhance its suitability for outbreak control and surveillance.

## Figures and Tables

**Table 1:** List of primers for RT-LAMP assay used in this study.

Primer	Length (bp)	5′Oligo Seq-3′
GI		
GI3017F3	18	GNTGGCAGGCCATGTTCC
GI3017B3-1	20	ACAGGATCCATTGCAAGAGG
GI3017B3-2	20	ACAGGTTCCATTGATATAGG
GI3017FIP-1	40	ACGAATTCGGGCAGRAGATYGC-
		CTGGATGCGMTTCCATGA
GI3017FIP-2	40	ACATAATCGGGCAGGAGATCGC-
		CTGGATGCGMTTCCATGA
GI3017BIP-1	40	TGATGATGGCGTCTAAGGACGC-
		TCCGGTACCAACTGACCA
GI3017BIP-2	40	TGATGATGGCGTCTAAGGACGC-
		TCTGGAACCAGCTGACCG
GI3017LF-1	21	ATCTCCTGTCCACAATCCGAG
GI3017LF-2	21	GTCCCCTGTCCACAAACTTAG
GI3017LB-1	17	GCGTGGATGGCGCYAGT
GI3017LB-2	17	ACATGGATGGCACCAGT
GII		
GII3017F3	20	GGTGGGATGGACTTTTACGT
GII3017B3-1	20	GCTCCAAAGCCATAACCTCA
GII3017B3-2	20	GTTCAAGAGCCATGACCTCA
GII3017FIP	39	GATTGCGATCGCCCTCCCAC-
		CAAGGCAGGAACCCATGTT
GII3017BIP-1	41	GAAGATGGCGTCGARTGACGC-
		TGTTGACCTCTGGTACGAGG
GII3017BIP-2	41	GAAGATGGCGTCGARTGACGC-
		TGTTACTTTCTGGCACGAGG
GII3017LF	22	GTCAGARAACCTCATCCACCTG
GII3017LB-1	20	CAACCCATCTGATGGGTCCG
GII3017LB-2	20	CGCTCCATCTACTGATGGTG

Degenerate bases used in primer sequences—R: A/G, Y: C/T, M: A/C, N: A/C/G/T.

## Data Availability

All data supporting the findings of this study are available within the manuscript and Supplementary Materials.

## Supplementary Materials

The following supporting information can be downloaded at: https://www.mdpi.com/article/10.3390/diagnostics15151868/s1, Figure S1: Validation with standard RNA; Figure S2: Sensitivity analysis of the target plasmid DNA; Figure S3: Field applicability of RT-LAMP assay under POCT platform; Table S1: List of pathogens used for specificity test.
