# Supplementary material for: Genogroup-Specific Multiplex Reverse Transcriptase Loop-Mediated Isothermal Amplification Assay for Point-of-Care Detection of Norovirus
Source: Diagnostics (Basel). 2025 Jul 25;15(15):1868. doi: 10.3390/diagnostics15151868 (PMC12345875; doi:10.3390/diagnostics15151868)
Supplement: Supplementary file 1 [file diagnostics-15-01868-s001.zip › diagnostics-3713735 Supplementary Table S1.pdf]

Supplementary Table S1: List of enteric pathogens used for the specificity of the developed RT-LAMP assays

Pathogen	Source (Strain No.)	Nucleic acid concentration
**Enteric Virus**		
Hepatitis E virus	ATCC-VR-3258SD	1×10^5copies/ul
Astrovirus	ATCC-VR-3238SD	1×10^5copies/ul
Sapovirus	ATCC-VR-3237SD	1×10^5copies/ul
Adenovirus type 40	KBPV-VR-6D	104.1ng/ul
Rotavirus A	KBPV-VR-47D	180.7ng/ul
Enterovirus D70	KBPV-VR-55D	135.3ng/ul
Enterovirus A71	KBPV-VR-56D	125.2ng/ul
Hepatitis A virus	KBPV-VR-78D	145.6ng/ul
**Enteric Bacteria**		
Salmonella Enterica	NCCP16521	222.8ng/ul
Salmonella Enterica	NCCP16937	193.2ng/ul
Escherichia coli	NCCP13905	299.1ng/ul
Escherichia coli	NCCP17169	565.1ng/ul
Escherichia coli	NCCP12579	259.5ng/ul
Staphylococcus aureus	MFDS2015478	30.4ng/ul
Staphylococcus aureus	MFDS2015479	29.4ng/ul
Yersinia Enterocolitica	MFDS2012373	257.3ng/ul
Yersinia Enterocolitica	MFDS2012374	320.4ng/ul
Listeria Monocytogen	MFDS2012614	10.3ng/ul
Listeria Monocytogen	MFDS2012615	11.3ng/ul
Shigella Flexneri	MFDS2013259	242.1ng/ul
Shigella Flexneri	MFDS2013260	64.5ng/ul
Shigella Flexneri	MFDS2013261	77.9ng/ul
Shigella Flexneri	MFDS2013262	205.4ng/ul
Vibrio Vulnificus	MFDS2014913	90.3ng/ul
Vibrio Vulnificus	MFDS2014914	141.8ng/ul
Vibrio Parahaemolyticus	MFDS2014883	250.5ng/ul
Vibrio Parahaemolyticus	MFDS2014884	238.0ng/ul
Escherichia coli	MFDS2014946	104.7ng/ul
Escherichia coli	MFDS2014947	90.0ng/ul
Escherichia coli	MFDS2015426	98.0ng/ul
Escherichia coli	MFDS2015427	99.9ng/ul
Escherichia coli	MFDS2014929	137.7ng/ul
Escherichia coli	MFDS2014931	395.9ng/ul
Escherichia coli	MFDS2014763	250.6ng/ul
Escherichia coli	MFDS2014764	366.9ng/ul
Escherichia coli	MFDS2014928	330.4ng/ul
Escherichia coli	MFDS2014932	251.7ng/ul
Escherichia coli	MFDS2014668	324.3ng/ul
Campylobacter Coli	MFDS2014643	54.0ng/ul

*ATCC: American Type Culture Collection, KBPV: Korea Bank for Pathogenic Viruses, NCCP: National Culture Collections for Pathogens; South Korea, MFDS: Ministry of Food and Drug Safety; South Korea. For nucleic acids, initial Concentration was shown, while final working concentration were used for viruses 1×10^4copies/ul and for bacteria 1ng/ul.
