# Supplementary figures and images for: Genogroup-Specific Multiplex Reverse Transcriptase Loop-Mediated Isothermal Amplification Assay for Point-of-Care Detection of Norovirus
Source: Diagnostics (Basel). 2025 Jul 25;15(15):1868. doi: 10.3390/diagnostics15151868 (PMC12345875; doi:10.3390/diagnostics15151868)

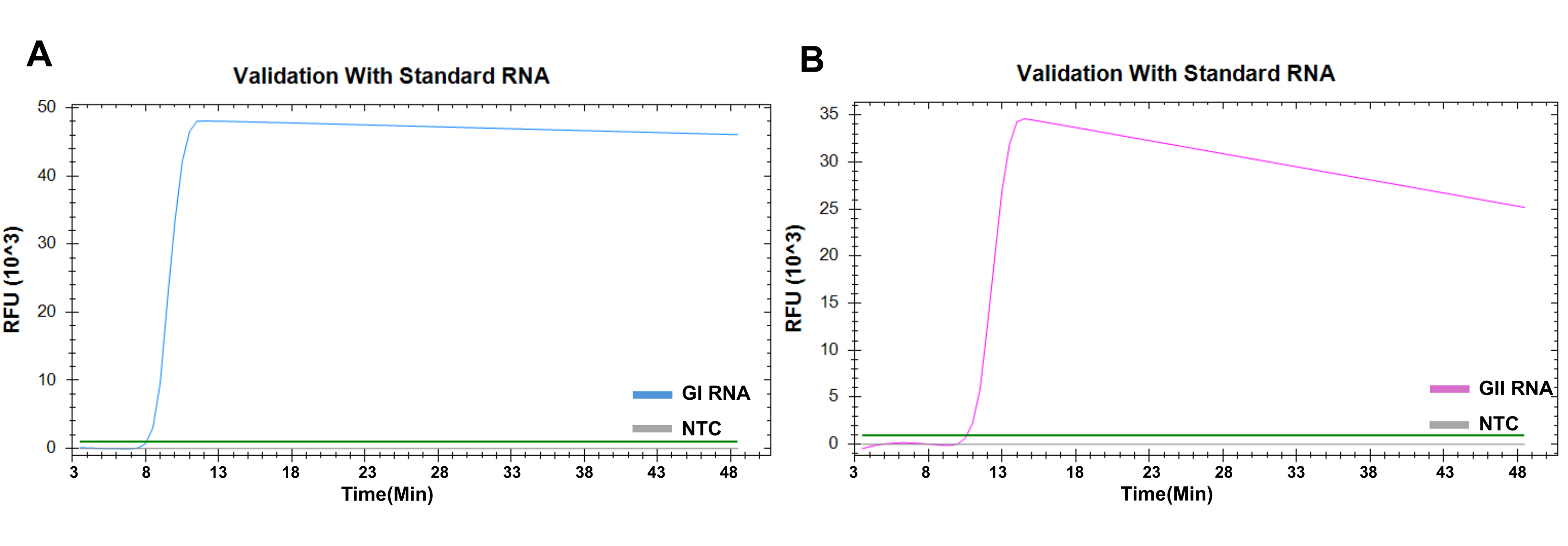

Supplement: Supplementary file 1 [file diagnostics-15-01868-s001.zip › diagnostics-3713735 Supplementary Figure S1.TIFF]

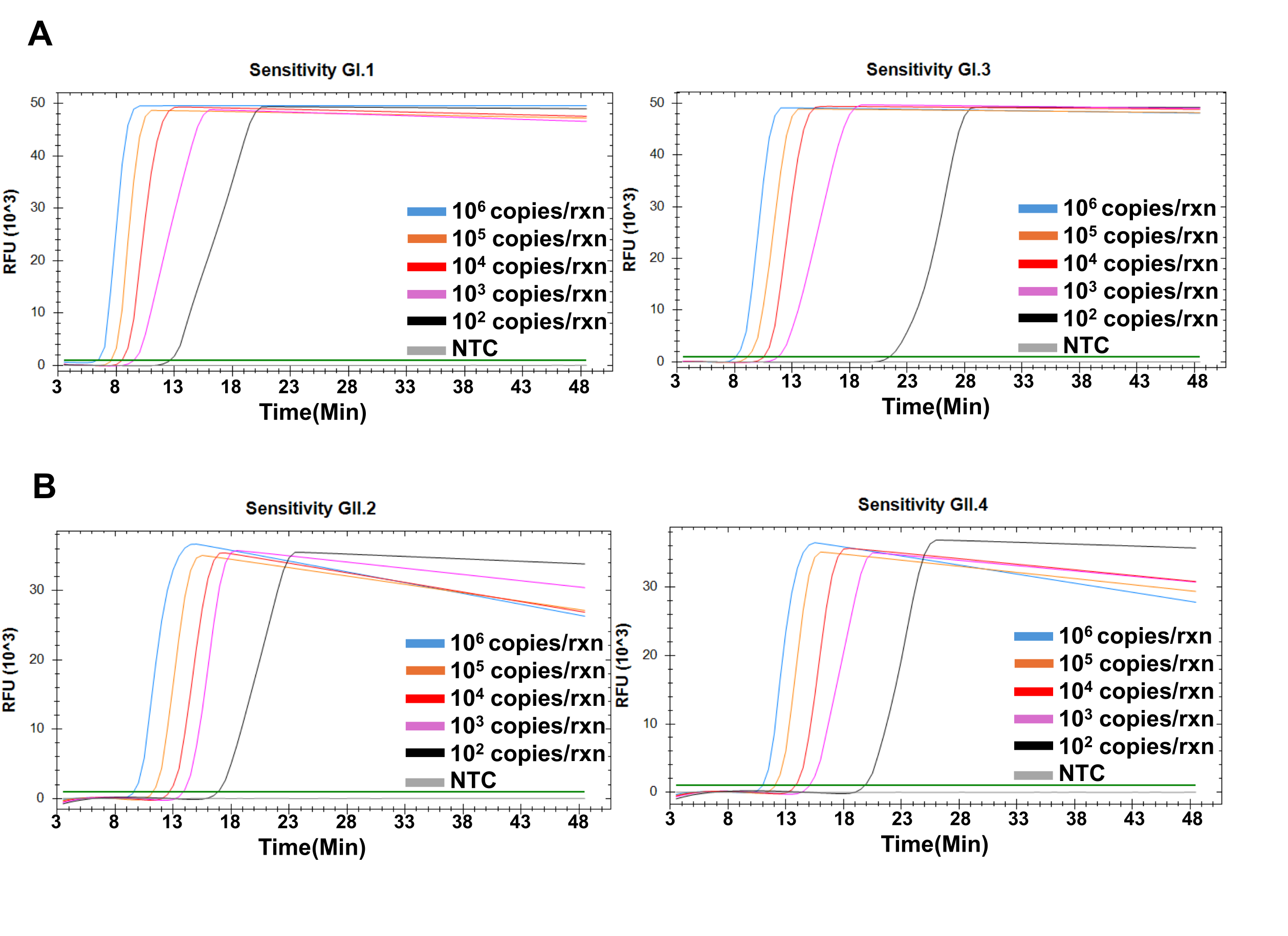

Supplement: Supplementary file 1 [file diagnostics-15-01868-s001.zip › diagnostics-3713735 Supplementary Figure S2.TIFF]

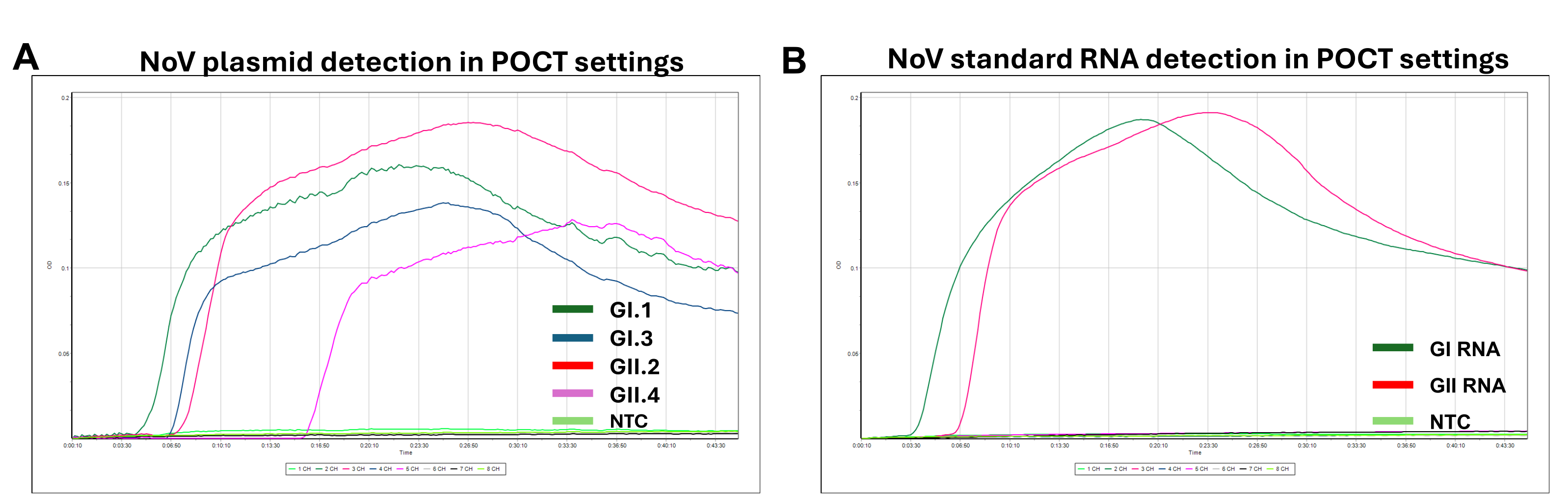

Supplement: Supplementary file 1 [file diagnostics-15-01868-s001.zip › diagnostics-3713735 Supplementary Figure S3.TIFF]
